# Air pollution modelling for birth cohorts: a time-space regression model

**DOI:** 10.1186/s12940-016-0145-9

**Published:** 2016-05-25

**Authors:** Elena Proietti, Edgar Delgado-Eckert, Danielle Vienneau, Georgette Stern, Ming-Yi Tsai, Philipp Latzin, Urs Frey, Martin Röösli

**Affiliations:** University Children’s Hospital (UKBB), University of Basel, Spitalstrasse 33 CH- 4056, Basel, Switzerland; Swiss Tropical and Public Health Institute (Swiss TPH), Socinstrasse 57, 4051, Basel, Switzerland; University of Basel, Basel, Switzerland; Division of Paediatric Pulmonology, Department of Paediatrics, Inselspital and University of Bern, Bern, Switzerland

**Keywords:** Air pollution, NO_2_, Exposure, Pregnancy, Birth cohort

## Abstract

**Background:**

To investigate air pollution effects during pregnancy or in the first weeks of life, models are needed that capture both the spatial and temporal variability of air pollution exposures.

**Methods:**

We developed a time-space exposure model for ambient NO_2_ concentrations in Bern, Switzerland. We used NO_2_ data from passive monitoring conducted between 1998 and 2009: 101 rural sites (24,499 biweekly measurements) and 45 urban sites (4350 monthly measurements). We evaluated spatial predictors (land use; roads; traffic; population; annual NO_2_ from a dispersion model) and temporal predictors (meteorological conditions; NO_2_ from continuous monitoring station). Separate rural and urban models were developed by multivariable regression techniques. We performed ten-fold internal cross-validation, and an external validation using 57 NO_2_ passive measurements obtained at study participant’s homes.

**Results:**

Traffic related explanatory variables and fixed site NO_2_ measurements were the most relevant predictors in both models. The coefficient of determination (R^2^) for the log transformed models were 0.63 (rural) and 0.54 (urban); cross-validation R^2^s were unchanged indicating robust coefficient estimates. External validation showed R^2^s of 0.54 (rural) and 0.67 (urban).

**Conclusions:**

This approach is suitable for air pollution exposure prediction in epidemiologic research with time-vulnerable health effects such as those occurring during pregnancy or in the first weeks of life.

**Electronic supplementary material:**

The online version of this article (doi:10.1186/s12940-016-0145-9) contains supplementary material, which is available to authorized users.

## Background

Air pollution exposure during early life, including pregnancy, may have consequences for the whole life and future generation as already demonstrated for smoking [1, 2]. Pregnancy is a vulnerable phase of life in which the fetus’ organs and systems develop in a specific order, time and speed. Depending on the period of pregnancy, air pollutants may reach and harm the fetus in different ways [3]. The time scale of these windows of vulnerability may be in the order of months or trimesters [4]. For instance, there is evidence for the effect of air pollution exposure during the last trimester of pregnancy on infant lung function [5–7], for infant mortality for respiratory reason due to exposure to air pollution during the first trimester [8], and for different lymphocyte distribution depending on the air pollution exposure for different trimesters [9, 10]. In the current literature, however, there is no consensus about the effect of air pollution on other birth outcomes such as birth weight or prematurity [11–15].

The assessment of exposure is a crucial step in the study of the potential adverse effects associated with air pollution. Errors in exposure measurements reduce the statistical power of a study [16] and bias the risk estimates to unity, both increasing the likelihood that real associations are not detected.

In birth cohort studies, models designed to accurately estimate individual traffic-related air pollution exposure for different biologically relevant time windows (i.e., during and after pregnancy) are therefore of extreme importance. A few birth cohorts have used dispersion models to estimate hourly or daily air pollution levels, and subsequently calculated exposure during pregnancy [13, 17, 18]. These models are very demanding in terms of data requirements and processing time, especially when the temporal and spatial resolution has to capture variation by season and within a few hundred meters. The easiest and most cost-effective way to estimate air pollution with the finest temporal resolution is to use data from fixed air quality monitoring (AQM) stations [19] with the disadvantage of having coarse spatial coverage. Inverse distance weighting and kriging may be used to model the spatial variability, though, depending on density of the monitors, complexity of topography, urbanization and meteorological conditions, these methods are often not sufficient to capture contrasts in exposures [19]. On the other hand, land use regression (LUR) models have been increasingly used to estimate long term exposure in cohort studies [20, 21]. In general LUR models focus on spatial variability over longer averaging periods, disregarding fine scale temporal variability, although attempts have been made to apply post-hoc temporal adjustments to LUR estimates by means of fixed air quality monitoring stations for birth cohort studies [15, 22–27]. However, this solution assumes no spatial changes in exposure patterns in time, which may not be applicable in some regions.

The aim of this study was to develop a model capturing the small scale spatial and temporal (monthly and biweekly) variation of nitrogen dioxide (NO_2_). The model integrates land use information, a dispersion model, temporal meteorological data, and measurements from the continuous air quality monitoring background station.

## Methods

### Air pollution measurements and study area

We used two different datasets of NO_2_ passive sampler measurements conducted continuously between 1998 and 2009. First, 24,499 biweekly NO_2_ measurements (consecutive 14-day exposure periods), sampled by the BECO (Berner Wirtschaft) at 101 sites located in a rural environment (i.e., the canton of Bern, area of 5959 km^2^ and includes several towns with less than 50,000 inhabitants), referred to as the BECO dataset. Second, 4350 consecutive monthly NO_2_ measurements from 45 sites situated in an urban environment (i.e., the city of Bern with 125,000 inhabitants and an area of 51.6 km^2^) conducted by the AFU (Amt für Umweltschutz Stadt Bern) and referred to as the AFU dataset. Both BECO and AFU are regulatory measurement networks designed to monitor air quality in the canton of Bern. The spatial distribution of the measurement locations is given in Additional file 1: Figure S1.

Site selection by BECO and AFU is aimed at monitoring the different environments generally present in the area: near highways (AFU 22 %, BECO 11 %), residential area near major roads within 100 m (AFU 36 %, BECO 38 %), rural area near major road within 100 m (BECO 24 %), urban setting with medium traffic (AFU 18 %) and low traffic (AFU 13 %), near industrial area (BECO 2 %), sites far from major road in residential (BECO 9 %) and rural areas (BECO 10 %), and urban and rural background (AFU 11 %, BECO 6 %). The BECO and the AFU analysed the passive diffusion samplers (Palmes tubes) in their own laboratories. The tubes were protected by a rain and wind shelter and placed at least 1.5 m above the ground. The precision in these measurements is ~5 % and the measurement of expanded uncertainty is below the recommended 25 % [28].

### Potential predictors of NO_2_

For each NO_2_ monitoring location we calculated spatial characteristics of the site (land use, roads, traffic, population, and annual NO_2_ levels from a dispersion model), meteorological conditions in the area during the time interval of the measurement, and NO_2_ concentrations from one representative continuous air quality monitoring station (Payerne, rural background site). A comprehensive overview of these predictors including the corresponding data source is provided in Additional file 1: Table S1.

#### Spatial predictors

We derived the geographic information system (GIS) variables using ArcGIS10.0, following the procedures in the ESCAPE project protocol [20, 29]. We obtained annual NO_2_ dispersion models (Pollumap, 400x400m resolution) for the whole of Switzerland from METEOTEST for every year between 2000 and 2007. We also obtained a traffic model (Gesamtverkehrsmodell – GMV Bern) for the whole road network of the canton of Bern developed by the Bau-, Verkehrs- und Energiedirektion des Kantons Bern (BVE) which models the annual average traffic of every road during workdays in 2007. The precision of the model is 5 % for the main road network and 8.8 % for the peripheral streets. Land use data for years 2000 and 2006 was issued by the Bundesamt für Umwelt (BAFU) and is based on the European CORINE classification. Population density was provided by the Amt für Geoinformation des Kantons Bern and was based on data collected in the year 2000. The altitude map as well as the road network (years 2000, 2004 and 2008) were derived from the Swisstopo database.

For land use, roads, traffic, and population density we considered several buffer sizes (50, 100, 200, 300, 500, and 1000 m) reflecting different dispersion patterns and scales of influence (local versus background sources) [30]. Source data for several spatial predictors were available for more than one time-point during the study period (Additional file 1: Table S1). In this situation, we linearly interpolated to estimate the predictors on an annual basis.

#### Temporal predictors

Temporal predictors included pollution measured at the representative continuous air quality monitoring (AQM) station reflecting the background level, and several meteorological parameters measured at local meteorological stations. For the background pollutant levels, we used the National Observational Network for Airborne Pollutants (NABEL) AQM station located in the countryside (Payerne). Measurements for temperature, pressure, humidity, wind speed, cloud coverage and solar radiation from local meteorological stations of the Federal Office of Meteorology and Climatology Meteoswiss were downloaded from the IDAweb data Portal (www.meteoswiss.ch). To assign meteorological conditions to all air pollution monitoring sites (AQM, BECO and AFU), we chose the nearest station considering topographical barriers. For the boundary layer height, as a proxy for inversion layer, we used 0.25° modelled grids from the European Centre for Medium-Range Weather Forecasts (ECMWF) ERA interim dataset. All the temporal predictors had a daily time resolution, which we averaged to correspond to the periods of the BECO and AFU NO_2_ measurements.

### Time-space exposure models development

We developed two distinct models: one for the rural and one for the urban environment. These regression models are based on the following formula:

$$ Log\left(N{O}_2\right)={\displaystyle \sum_{i=1}^n{\beta}_i{X}_{is}+}\kern0.5em {\displaystyle \sum_{j=1}^m{\beta}_j{X}_{jt}} $$

Where: *ß* is a regression coefficient, *Xs* is a spatial covariate, n is the number of measurement locations, *Xt* is a temporal covariate, and *m* is the number of observation periods. For model development we applied a log transformation to the dependent variable to take into account the skewed data distribution. No intercept was considered because we included background NO_2_ levels from the dispersion model. Given that the R^2^ is not provided in the regression output when the intercept is suppressed (i.e., forced through the origin), we manually calculated the coefficient of determination (R^2^). To select the predictors, we first grouped the variables thematically based on prior knowledge: various types of land use, traffic, roads, topography, NO_2_ from dispersion model, NO_2_ from continuous AQM, and meteorology (Additional file 1: Table S1). Within some groups, variables were computed for different buffer sizes (e.g., 50, 100, 200, 300, 500, and 1000 m) and several characteristics (e.g., roads represented by distance to street and street density within buffers). We first built a base model including one variable per thematic group chosen a priori according to previous studies. The final variable selection was obtained by using an iterative variable selection procedure combining supervised stepwise forward (bivariate models) and stepwise backward regression to: (a) evaluate the relevance of a thematic group, and (b) select variable(s) per retained thematic group [20, 29]. To determine the most suitable predictor or combination of predictors within a thematic group we tested different options in turn (i.e., buffer size, transformations, splines) and selected the best on the basis of physical/chemical plausibility as well as the R^2^ of the model and R^2^ from ten-fold cross-validation (see section 2.4). For differences in R^2^ less than 1 %, we prioritised small number of variables, similar buffer sizes, the most linear dependency as possible, and no transformation of the variable. This process was reiterated until the model converged. Finally we tested space-time interactions.

### Internal cross-validation

For the rural and the urban model we performed ten-fold cross-validations. This involves using 90 % of the dataset to fit the model, having fixed the variables of the model but allowing the coefficients to change. We then used the derived model to estimate the remaining 10 % of the dataset. We repeated this process ten times in order to estimate all observations once. Finally we compared the estimated with the measured values assessing Pearson r, R^2^ and root mean square errors (RMSE). Validation results were calculated for both the log transformed scale and real concentrations (i.e., exponentiated the predicted values and compared with the measured concentrations).

### External validation

Between years 2010 and 2012 we performed 57 NO_2_ biweekly measurements at the home addresses of a subsample of our BILD (Basel-Bern Infant Lung Development) birth cohort study participants for validation purposes (referred to as the “study dataset”) [31]. The study was approved by the ethics committee of the Canton of Bern, Switzerland. Informed consent was provided by parents or caregivers. All parents who enrolled in the study from 2010 until end of 2012 were instructed by the study nurses to place a passive Passam sampler outside their home for a 14-day period within the first month after their child’s birth. The sites were thus scattered over the Bernese region: one third in the city of Bern and two thirds in the Canton of Bern. For this study dataset, we used samplers provided and analysed by Passam AG, Männedorf, Switzerland. The precision of Passam tubes is comparable to Palmes tubes [28].

For the external validation, depending on the location of the study participants, we used the urban or rural model to estimate the NO_2_ concentrations during the measurement period. We assessed the same parameters as for the internal cross-validation, in addition to Kappa statistics comparing quartiles of measured and estimated values, and conducted a descriptive analysis to evaluate factors possibly related to the modelling residual (error).

## Results

**Fig. 1:**
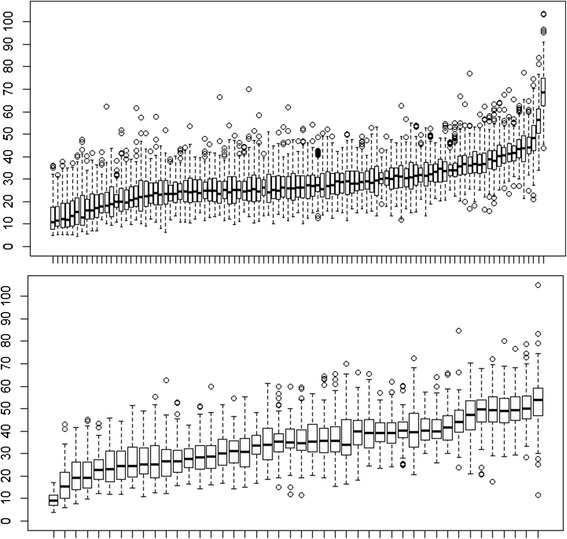
Descriptive summary of NO_2_ measurements in the rural region (*top*) and urban area (*bottom*) for period 1998 to 2009 for each measuring site in μg/m^3^ (Box plots for each monitoring location showing median, 1st and 3rd quartile of the measurements for each site, ordered by average NO_2_ concentration)

The NO_2_ concentration measured at each site in the BECO and AFU datasets is shown in Fig. 1. A similar range in concentrations was measured in both the rural region and the urban area (4 to 103 μg/m^3^), with a median of 27 μg/m^3^ in the rural region and of 35 μg/m^3^ in the city. The difference between lowest and highest site-specific NO_2_ annual mean levels was 61 μg/m^3^ in the rural region, and 45 μg/m^3^ in the urban area. The annual trend in the rural region was 1 μg/m^3^/year decrease from 1999 to 2001. After 2001 the average annual NO_2_ concentration was stable at 28 μg/m^3^. The temporal pattern for a sample of the AFU sites indicates temporal variations in the spatial pattern of NO_2_ over the study area (Additional file 1: Figure S2).

The final rural model included 17 predictors plus 1 interaction term (Table 1) and explained 63 % of the variability in the NO_2_ measurements (R^2^). The urban model included 13 predictors and 2 interaction terms (Table 2), explaining 54 % of the NO_2_ variability. Altitude was not considered for the urban model, as there is little variability in elevation within the city. All predictors were statistically significant (*p*-value <0.001) even in the cross-validation process, and were not strongly auto-correlated (Variance Inflation Factors - VIF <10 [32]) (Additional file 1: Table S2).

**Table 1:** Final model for the rural region

Variables	Percentile			Estimate per IQR^a^	95 % CI lower	95 % CI upper	Cumulative Adj. R^2^
	25	50	75				
Total length of major roads in 100 m buffer * season^b^	0	294	563	−0.363	−0.382	−0.345	0.278
Vehicles in 50 m buffer *N*	67068	862600	1730503	0.146	0.141	0.150	0.334
High density residential land use in 200 m buffer *percent area*	0	0	0	0.410	0.389	0.430	0.372
Log (NO_2_ from AQM Payerne) *log(NO*_*2*_ *concentration)*	2.28	2.62	2.98	0.250	0.239	0.262	0.406
Log (NO_2_ from dispersion model) *log(NO*_*2*_ *concentration)*	2.94	3.08	3.21	0.028	0.022	0.035	0.510
Total length of major roads in 100 m buffer *m*	0	197	238	0.474	0.456	0.492	0.563
Season (summer = 1, mid-season = 2, winter = 3)^b^	1	2	3	0.181	0.158	0.203	0.578
Sqrt(Traffic in the nearest road) *sqrt(N)*	0.0	12.5	67.3	0.098	0.092	0.104	0.591
Industrial land use in 300 m buffer *percent area*	0	0	0	0.321	0.300	0.342	0.603
Population in 100 m buffer *N*	13.5	103.3	156.1	0.051	0.045	0.057	0.611
Linear time trend *year*	2001.7	2004.3	2007.1	0.529	0.499	0.558	0.614
Linear time trend ^2 *(year^2)*	2001.7^2^	2004.3^2^	2007.1^2^	−0.559	−0.593	−0.525	0.618
Total length of major roads in 1000 m buffer *m*	0	197	238	0.038	0.030	0.046	0.622
Temperature *Celsius*	3.65	9.75	16.14	−0.102	−0.115	−0.090	0.625
Altitude *m*	460	535	561	−0.032	−0.036	−0.028	0.628
Low density residential land use in 200 m buffer *percent area*	0.301	0.999	0.999	0.108	0.094	0.122	0.631
Boundary layer height *m*	126.2	319.7	656.2	−0.022	−0.030	−0.014	0.632
Total length of major roads in 500 m buffer *m*	0	197	238	0.012	0.004	0.020	0.632

Model developed without an intercept term. The R^2^ is not provided in the regression output when the intercept is suppressed; we thus manually calculated the R^2^. The predictors are ordered per decreasing relevance on the basis of incremental R^2^. All *p*-values were <0.001

* indicates multiplication of variables

^a^For land use data (high and low density residential land use and industrial land use) we report the estimate per increase from 0 to 100 % of used area instead of per increase of IQR because data distribution is skewed and IQR would be 0

^b^Season categorised as 1: summer (May to August), 2: mid-season (March, April, September, October), 3: winter (November to February)

**Table 2:** Final model for the urban area

Variables	Percentile			Estimate per IQR^a^	95 % CI lower	95 % CI upper	Cumulative Adj. R^2^
	25	50	75				
Sqrt (vehicles in 100 m buffer) * season^b^	1728	3696	6117	−0.219	−0.265	−0.172	0.291
Log (NO_2_ from dispersion model) *log(NO*_*2*_ *concentration)*	3.21	3.28	3.37	0.052	0.039	0.065	0.341
Log (NO_2_ from AQM Payerne) *log(NO*_*2*_ *concentration)*	2.3	2.68	3.03	0.216	0.181	0.252	0.372
Sqrt (vehicles in 100 m buffer)	1391	1997	3074	0.404	0.362	0.446	0.437
Log(1/distance to the nearest major road) *log(1/m)*	−4.08	−2.95	−2.61	0.163	0.144	0.181	0.470
Linear time trend *year*	2002.6	2005.2	2007.7	0.477	0.387	0.567	0.488
Season (summer = 1, mid-season = 2, winter = 3)^b^	1	2	3	0.191	0.118	0.264	0.499
Industrial land use in 300 m buffer *percent area*	0	0	0.237	0.436	0.384	0.487	0.506
Population in 100 m buffer *N*	0.95	141	323	0.118	0.097	0.139	0.514
(Total length of major roads in 100 m buffer)^2 *(m^2)*	26931	48969	147510	0.296	0.259	0.334	0.519
Total length of major roads in 100 m buffer *m*	164	221	384	−0.414	−0.472	−0.356	0.534
Linear time trend ^2 *(year^2)*	2002.6^2^	2005.2^2^	2007.7^2^	−0.462	−0.563	−0.36	0.540
Temperature *Celsius*	3.4	9.05	15.59	−0.081	−0.126	−0.035	0.540
(Boundary layer height)^2 *(m^2)*	16723	79082	359729	−0.013	−0.024	−0.002	0.541
Total length of major roads in 100 m buffer * temperature	0	1485	3807	0.034	0	0.069	0.541

Model developed without an intercept term. The R^2^ is not provided in the regression output when the intercept is suppressed; we thus manually calculated the R2. The predictors are ordered per decreasing relevance on the basis of incremental R^2^. Most *p*-values were <0.001; *p*-value for “Total length of major roads in 100 m buffer * temperature” was <0.05

* indicates multiplication of variables

^a^For land use data (high and low density residential land use and industrial land use) we report the estimate per increase from 0 to 100 % of used area instead of per increase of IQR because data distribution is skewed and IQR would be 0

^b^Season categorised as 1: summer (May to August), 2: mid-season (March, April, September, October), 3: winter (November to February)

In order to compare the relevance of the various predictors in our study areas, model coefficients in Tables 1 and 2 are expressed per interquartile (IQR) change of the predictor variable and ordered per contribution of R^2^ to the whole model. In both models the traffic-related predictors occupied the highest positions in the models, and the most relevant predictor in both models was the interaction between season and a proxy for traffic. Another very relevant (temporal) predictor in both models was the NO_2_ concentration at the rural background site representing temporal variation of NO_2_ in the study areas. The explanatory power of the NO_2_ levels from the dispersion model, representing spatial variability of background concentrations, was somewhat lower in both models than the fixed site NO_2_ measurements. Both models included population density, and the rural model additionally included a residential land use variable. Residential land use, however, did not improve the performance of the urban model and was not retained in the model. In both models year was treated as polynomial (linear and square term) as the splines showed a non-linear correlation. Both a rural and urban model containing only spatial predictors explained ~40 % of the NO_2_ variability; temporal predictors alone explained 22 % of the variability in the rural region and 13 % of the variability in the urban area.

For the rural model, the R^2^ based on the log transformed NO_2_ measurements was 0.63 and for the untransformed measured concentration was 0.61 (Table 3). The same R^2^s were obtained for the ten-fold internal cross-validation indicating robust coefficient estimates. For the urban model the R^2^s were somewhat lower but again, identical for the internal cross-validation. The Bland-Altman plots of the internal cross-validation show a negative slope with an over prediction of the lower values (Additional file 1: Figure S3).

**Table 3:** Performance and validation of the final models

Area	Evaluation	Pearson r		R^2^		RMSE
		Log (μg/m^3^)	μg/m^3^	Log (μg/m^3^)	μg/m^3^	μg/m^3^
Rural	Model	0.79	0.78	0.63	0.61	5.86
	Internal cross-validation	0.80	0.78	0.63	0.61	5.86
	External validation	0.77	0.82	0.58	0.68	3.21
Urban	Model	0.74	0.67	0.54	0.45	6.96
	Internal cross-validation	0.74	0.67	0.54	0.45	6.96
	External validation	0.82	0.83	0.67	0.69	3.35

Internal cross-validation was based on ten-fold cross-validation, and external validation used the study dataset. We compared measured and predicted values on the log scale, on which the models were developed, and as concentrations by exponentiating the predictions. The root mean square errors (RMSE) are derived from the comparison of NO_2_ concentrations only

The study dataset for external validation recorded 57 NO_2_ values ranging from 4 to 33 μg/m^3^ (median 15 μg/m^3^). Thirty eight measurements were performed in the rural region (median 12.2 μg/m^3^ [IQR 7.9–21]), and 19 measurements were performed in the urban area (median 24.1 μg/m^3^ [IQR 14.8–28.2]). The samples were uniformly distributed across the different seasons. We observed that 26 parents placed the samples in the backyard. Based on an analysis of the model residuals for backyard measurements compared to the other outdoor measurements, backyard measurements were corrected by a factor of 1.104 in urban settings and 1.275 in rural settings.

After backyard correction, the external validation of the urban model had comparable Pearson r, R^2^, and RMSE to the model itself and the internal cross-validation (Table 3). The urban model performed better in the external validation, with higher R^2^ and a remarkably lower RMSE. The Bland-Altman plot of the study dataset, comparing measured and predicted values for the rural or urban model depending on the location of measurement, showed no evident slope but still an overestimation of 2.1 μg/m^3^ (Additional file 1: Figure S4).

For the external validation, an exposure assessment based on quartile resulted in a weighted Kappa coefficient of 0.671 between predicted and measured NO_2_ levels (Additional file 1: Table S3).

## Discussion

The rural and urban models that we developed are based on biweekly and monthly measurements and have been externally validated. We found that the most important predictors, as indicated by the IQR change of the predictor variable, in both models were those related to traffic. A finding that may be of particular interest for policy makers is that the models show the overwhelming impact of the traffic-related predictors on air pollution over the temporal component, and the data did not show any downward trend over the last years.

We showed the importance of having both a temporal and spatial component in such an air pollution exposure model. Estimation based on temporal components alone would only explain 12 to 22 % of the NO_2_ variability, and the spatial component alone only 40 %. Combined, however, for log transformed models we reached an R^2^ of 0.63 in the rural setting and 0.54 in urban areas. It seems that the duration of the NO_2_ measurement plays a role for the temporal R^2^. For the rural model with biweekly measurements a larger proportion of the variance is explained by temporal predictors compared to the urban model, which is based on monthly measurements. In general, however, the R^2^s of both models are comparable to annual LUR models for a wide range of European cities (ESCAPE study, 36 study areas, R^2^: 0.31–0.87) [20]. The comparison is even better when we average our data to generate annual LUR models. Model performance, as indicated by R^2^, for annual models using only the spatial predictors in our final model ranged from 64 to 75 % for rural and 48 to 74 % for the urban area (data not shown).

Our approach has several clear strengths compared to previous models. The first is the number of available temporal observations. Previously published models were often limited to annual averages based typically on three measurement periods per year (one per season) [21, 33, 34], whereas our model was developed using 12 to 26 measurements per year over a period of more than ten years. This wealth of data enabled us to develop a more robust model which can be used predictively to assign exposures to cohort studies. A similar network of passive samplers was recently used to develop 14-day NO_2_ concentration maps for the city of Zurich, Switzerland [35]. That study, however, aimed more generally at air quality assessment for cities rather than prediction for individuals during critical time windows.

Given the number of predictor variables in the model, one concern is that model might be over-specified. However, we can rule this out because of similar results for the internal cross-validation and external validation. Nevertheless, 10 variables in the rural model and 5 in the urban model added only 3 % to the explained variance. To evaluate the impact of this on the estimates we tested a model without these variables and found that R^2^ in the external validation decreased from 0.63 to 0.60 (rural model) and from 0.54 to 0.49 (urban model). We further found that the degree of overestimation increased from 2.11 μg/m^3^ to 3.29 μg/m^3^. Since the dataset is very large, and the extra work to include these variables is negligible, we opted to aim at the best model which explains most of the variance. Using a large number of temporal measurements also minimised the likelihood of over-fitting the model [36]. An internal cross-validation that does not agree with the original model would be an indication for this kind of problem. In our study the ten-fold internal cross-validation showed the same values of performance (R^2^, Pearson r and RMSE) as the original model, attesting to the stability of the model. In our estimation process we did not account for temporal and spatial correlation of the measurements. This affects the confidence intervals of the model coefficients but is unlikely to produce a bias. Exposure prediction is based on the central estimates only.

We were able to validate the model using an external dataset with measurements performed in the same area but at different sites and in a different time frame (1998–2009 for the model training measurements vs. 2010–2012 for the external validation). The advantage of the study dataset is that the sites reflect the residence (home location) of study participants, thus actual exposure locations. In contrast, the AFU and BECO measurement sites are not expected to fully reflect the spatial distribution and variation in exposures at the home addresses of our BILD birth cohort participants because the networks were designed to over-represent near street environments. We found that the predictions of the external dataset were overestimated. A part of this overestimation could be attributed to the fact that study dataset measurements were partly done in the backyard of the residence. After applying a backyard correction, an overestimation of ~2.1 μg/m^3^ was still seen. Possible reasons for this are the known overestimation of lower values of such kind of regression models. Since the study participants generally do not live in air pollution hot spots, such as near highways, the dataset is situated in the lower range where we observe this systematic error as a consequence of regression to the mean. Another explanation could be the differences in sampling methodology (Passam vs Palmes tubes), however this is unlikely as the literature to date reports good agreement between the two equipment types [28]. A further consideration could be the NO_2_ trend over time. The training dataset encompassed the years 1998 to 2009 and the study dataset covered 2010 until 2012. A decrease in NO_2_ levels in recent years [37, 38] could explain an overestimation of values by the model. However, a decrease in overall NO_2_ levels has not been observed since 2001 in our dataset. Therefore this hypothesis is unlikely. Most importantly, however, we found that the prediction of the study dataset using our models was reasonable. The external validation R^2^ based on the rural measurements was the same as the model R^2^, while for urban measurements the external validation R^2^ was higher, probably due to the restricted range.

Some birth cohorts in Europe are using temporally adjusted land use regression models based on one or a few AQM stations [22, 26, 39]. They apply a global adjustment, thus the spatial pattern remains the same across cohort period which is not realistic. Given that we have a complex topography and temporal variation of the spatial pattern (Additional file 1: Figure S2) in our study area, a global adjustment would not suffice. In comparison to these earlier studies, our approach more realistically and systematically models the spatial and temporal variability of air pollution exposures. Our results also suggest that the spatial component alone is unlikely to reflect well the variations in air pollution at shorter time periods, such as those needed for birth cohorts.

## Conclusions

Our model could predict quite well biweekly or monthly NO_2_ levels at independent measurement locations. As such, it will be used to predict NO_2_ exposure during pregnancy for various time intervals during and shortly after pregnancy to support the investigation of subsequent health effects. To this end, we can estimate exposure for individual cohort participants by specific time windows (e.g., trimesters, full pregnancy, or 1^st^ year of life). Thus, our approach is an exemplary tool for air pollution exposure prediction in time-sensitive epidemiologic research with seasonally-vulnerable health effects such as the effects occurring during pregnancy.

## Additional file

Additional file 1: Figure S1. Spatial distribution of the BECO (rural) and AFU (urban) measurement locations in the canton of Bern, displayed on background NO_2_ from the 2007 dispersion model. Figure S2. NO2 levels measured in a sample of urban monitoring sites during the year 2007. Figure S3. Internal validation Bland-Altman plot of predicted and measured values in the rural region (top) and urban area (bottom) in the log scale. Figure S4. Bland-Altman plot for external validation in μg/m^3^ (rural or urban model without intercept, corrected for backyard measurements). Table S1. Potential predictors of NO_2_. Table S2. Variance Inflation Factors (VIF) of main predictors in the rural and urban model. Table S3. Kappa statistics for External validation – measured vs estimated concentration in quartiles. (DOCX 1047 kb)

## Abbrievations

AFU: Amt für Umweltschutz Stadt Bern, i.e., monitoring network in city of Bern
AQM: air quality monitoring
BECO: Berner Wirtschaft, i.e., monitoring network in rural Bern
BILD: Basel-Bern Infant Lung Development
CORINE: Coordination of information on the environment, i.e., land cover data
ECMWF: European Centre for Medium-Range Weather Forecasts
ESCAPE: European Study of Cohorts for Air Pollution Effects
GIS: geographic information system
IQR: interquartile range
LUR: land use regression
NABEL: National Observational Network for Airborne Pollutants
NO_2_: nitrogen dioxide
R^2^: coefficient of determination
RMSE: root mean square error
VIF: variance inflation factor
