# Supplementary material for: Air pollution modelling for birth cohorts: a time-space regression model
Source: Environ Health. 2016 May 25;15:61. doi: 10.1186/s12940-016-0145-9 (PMC4881180; doi:10.1186/s12940-016-0145-9)
Supplement: Additional file 1: — Figure S1. Spatial distribution of the BECO (rural) and AFU (urban) measurement locations in the canton of Bern, displayed on background NO2 from the 2007 dispersion model. Figure S2. NO2 levels measured in a sample of urban monitoring sites during the year 2007. Figure S3. Internal validation Bland-Altman plot of predicted and measured values in the rural region (top) and urban area (bottom) in the log scale. Figure S4. Bland-Altman plot for external validation in μg/m3 (rural or urban model without intercept, corrected for backyard measurements). Table S1. Potential predictors of NO2. Table S2. Variance Inflation Factors (VIF) of main predictors in the rural and urban model. Table S3. Kappa statistics for External validation – measured vs estimated concentration in quartiles. (DOCX 1047 kb) [file 12940_2016_145_MOESM1_ESM.docx]

Additional file 1

**Air pollution modelling for birth cohorts: a hybrid time-space regression model**

**Elena Proietti^1,4^, Edgar Delgado-Eckert^1^, Danielle Vienneau^2,3^, Georgette Stern^4^, Ming Tsai^2,3^, Philipp Latzin^1^, Urs Frey^1^, Martin Röösli^2,3^**

*^1^ University Children’s Hospital (UKBB), University of Basel, Switzerland*

*^2^ Swiss Tropical and Public Health Institute (Swiss TPH), University of Basel Switzerland*

*^3^ University of Basel, Basel, Switzerland*

*^4^ Division of Paediatric Pulmonology, Department of Paediatrics, Inselspital and University of Bern, Switzerland*

Figure S1. Spatial distribution of the BECO (rural) and AFU (urban) measurement locations in the canton of Bern, displayed on background NO_2_ from the 2007 dispersion model


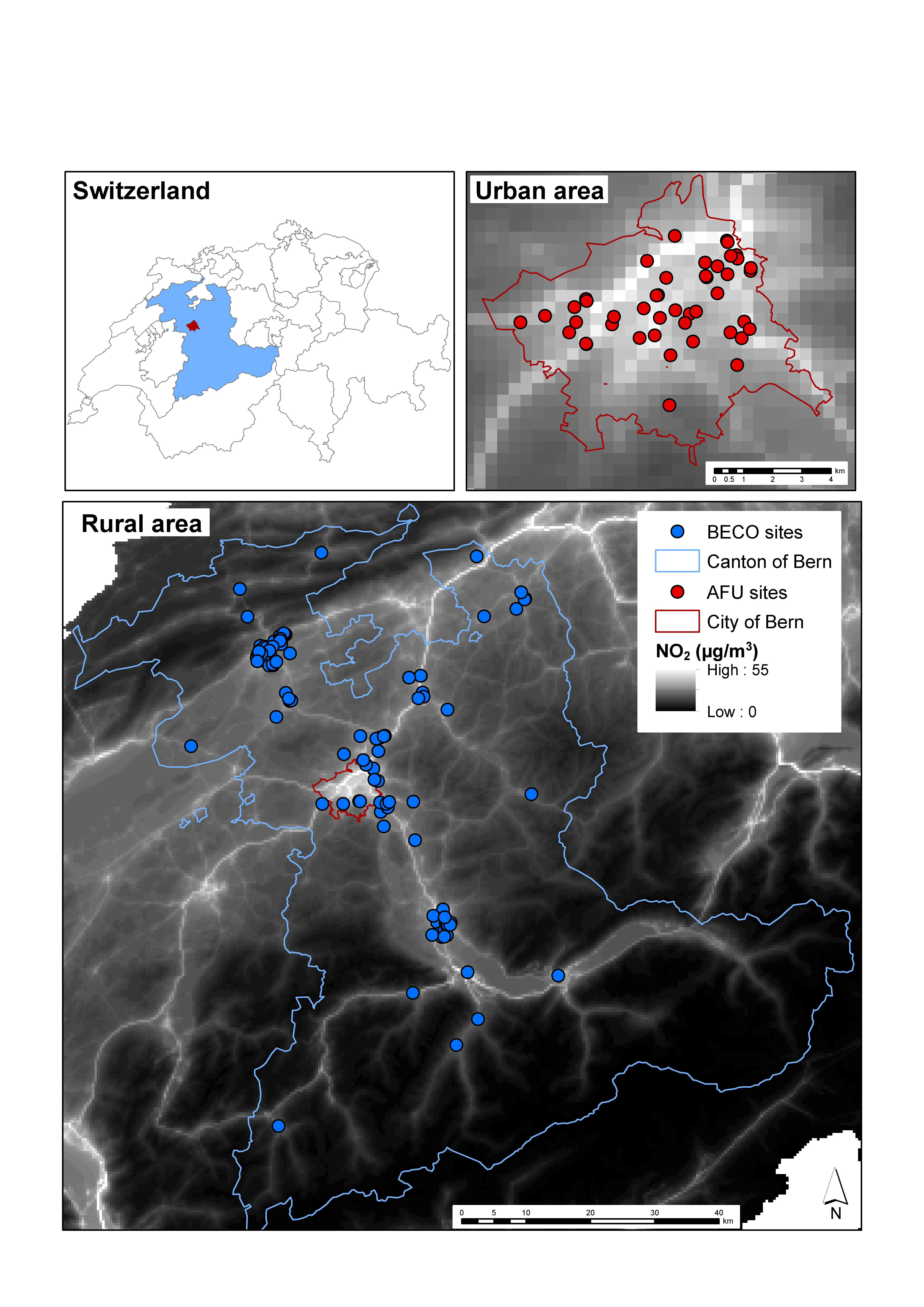


Figure S2: NO2 levels measured in a sample of urban monitoring sites during the year 2007


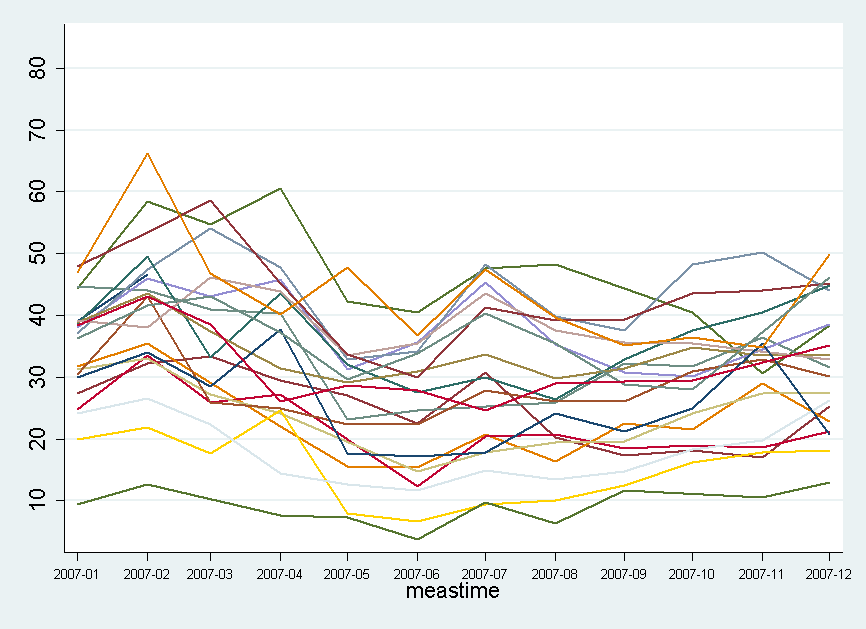


Figure S3. Internal validation Bland-Altman plot of predicted and measured values in the rural region (top) and urban area (bottom) in the log scale


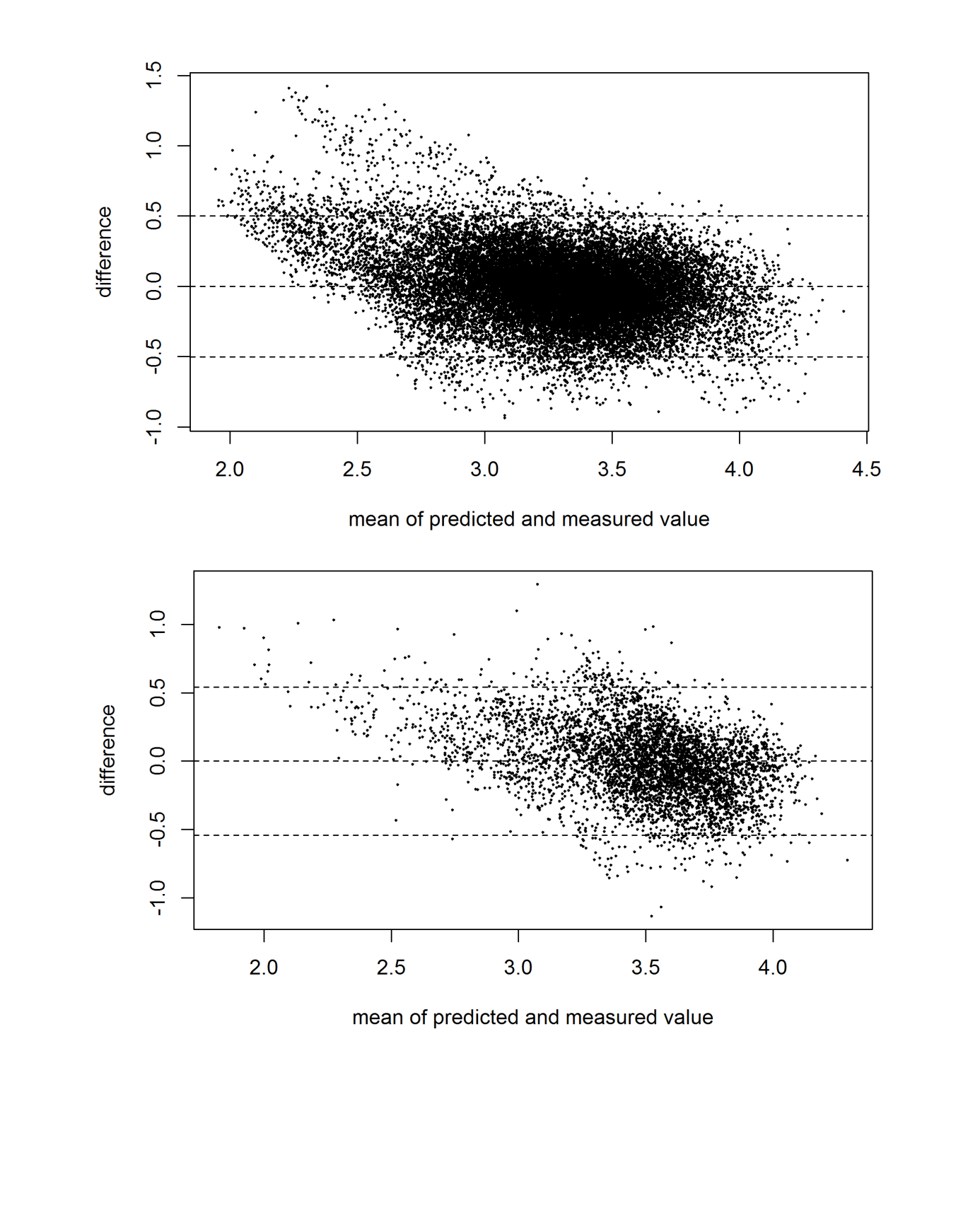


Figure S4. Bland-Altman plot for external validation in µg/m^3^

(rural or urban model without intercept, corrected for backyard measurements)


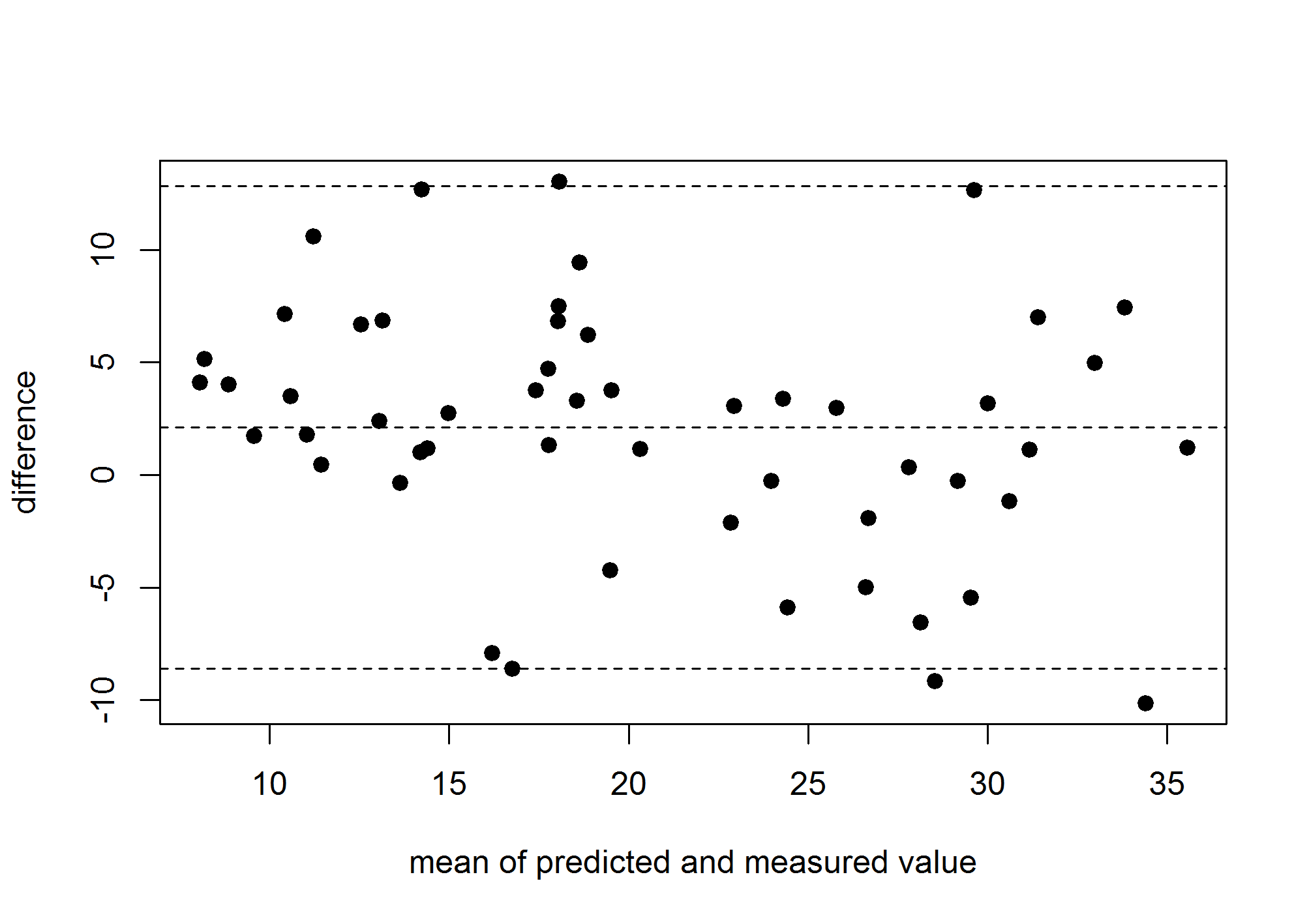


Table S1. Potential predictors of NO_2_

| **Thematic Group** | **Source of data** | **Predictor variable** | **Unit** | **Buffer^a^** | **Direction of Effect** |
| --- | --- | --- | --- | --- | --- |
| **Space-related predictors** | | | | | |
| Land use | CORINE classification  (Bundesamt für Umwelt - BAFU))  years 2000 and 2006 | High density residential use | Proportion | yes | + |
|  |  | Low density residential use | Proportion | yes | + |
|  |  | Industry | Proportion | yes | + |
|  |  | Agriculture | Proportion | yes | + |
|  |  | Urban green, Semi-natural and forested areas | Proportion | yes | - |
| Population | Population density  (Amt für Geoinformation des Kantons Bern)  Year 2000 | Number of inhabitants | Number | yes | + |
| Topography | Altitude  (DHM25 map - Swisstopo) | Altitude | m | no | - |
| Traffic | Regional traffic model network  (Bau-, Verkehrs- und Energiedirektion des Kantons Bern (BVE))  Year 2007 | Traffic intensity on nearest road | Veh.day^-1^ | no | + |
|  |  | Total traffic load of all roads in a buffer (sum of (traffic intensity * length of road segment)) | Veh.day^-1^m | yes | + |
|  |  | Product of traffic intensity on nearest road and inverse of distance to the nearest road | Veh.day^-1^m^-1^ | no | + |
| Roads | National road network  (VECTOR25 Swisstopo)  Years 2000, 2004 and 2008 | Distance to the nearest first class road | m,m^-1^, m^-2^ | no | + |
|  |  | Distance to the nearest highway | m,m^-1^, m^-2^ | no | + |
|  |  | Distance to the nearest major road (highway or first class road) | m,m^-1^, m^-2^ | no | + |
|  |  | Total road length in a buffer | m | yes | + |
|  |  | Total first class road length in a buffer | m | yes | + |
|  |  | Total highway length in a buffer | m | yes | + |
|  |  | Total major road length in a buffer (highway plus first class road) | m | yes | + |
| Dispersion model | Annual NO_2_ national maps 400m grids  (Bundesamt für Umwelt (BAFU) and Meteotest)  All years between 2000 and 2007 | Modelled NO_2_ annual average | µg.m^-3^ | no | + |

| **Thematic Group** | **Source of data** | **Predictor variable** | **Unit** | **Buffers^a^** | **Effect** |
| --- | --- | --- | --- | --- | --- |
| **Time-related predictors** | | | | | |
| Season |  | Summer, midseason, winter |  | NA | + |
| NO_2_ from continuous AQM^b^ | Nationales Beobachtungsnetz für Luftfremdstoffe (NABEL) | AQM station of Payerne (rural background) | µg.m^-3^ | NA | + |
| Meteorology | Meteo stations of the Federal Office of Meteorology and Climatology Meteoswiss  IDAweb data Portal  [www.meteoswiss.ch](http://www.meteoswiss.ch) | Precipitation | mm | NA |  |
|  |  | Temperature 2m above soil | °c | NA | - |
|  |  | Relative humidity | % | NA |  |
|  |  | Cloud cover | % | NA | + |
|  |  | Wind speed | m/s | NA | - |
|  |  | Atmospheric pressure | hPa | NA |  |
|  |  | Global radiation | W/m² | NA | - |
| Proxy for Air mixing layer | ERA interim dataset - The European Centre for Medium-Range Weather Forecasts (ECMWF)  <http://apps.ecmwf.int/datasets/data/interim_full_daily/> | Modelled boundary layer height | m | NA | - |

Notes:

The direction of effect indicates whether we expected a positive or negative correlation of the predictor with the outcome (NO_2_ level)

a. Buffers of 50, 100, 200, 300, 500 and 1000 meters around the NO_2_ measuring site. NO2 concentrations typically decay to background levels within a few hundred metres of the source.^1-3^ Correlations between the 300m and 500m buffers were high (r= 0.83 ̶ 0.98), thus a 400m buffer was not included. NA: Not Applicable, AQM: Air Quality Monitor.

b. We evaluated the available background monitoring stations in the canton of Bern. Payerne was selected based on having the most complete time series as well as high correlations with the other background sites. Furthermore, Payerne is the most remote background monitoring location in the canton.

References:

1. Gilbert NL, Woodhouse S, Stieb DM, Brook JR. Ambient nitrogen dioxide and distance from a major highway. Sci Total Environ 2003;312:43–6.

2. Pleijel H, Pihl Karlsson G, Binsell Gerdin E. On the logarithmic relationship between NO2 concentration and the distance from a highroad. Sci Total Environ 2004;332:261–4.

3. Roorda-Knape MC, Janssen NAH, De Hartog JJ, van Vliet PHN, Harssema H, Brunekreef B. Air pollution from traffic in city districts near major motorways. Atmos Environ 1998;32(11):1921–30.

Table S2. Variance Inflation Factors (VIF) of main predictors in the rural and urban Model

| **Rural Model** | **VIF** |
| --- | --- |
| Vehicles in 50m buffer *N* | 2 |
| High density residential land use in 200m buffer *percent area* | 2 |
| Log (NO_2_ from AQM Payerne) *log(NO_2_ concentration)* | 5 |
| Log (NO_2_ from dispersion model) *log(NO_2_ concentration)* | 2 |
| Total length of major roads in 100m buffer *m* | 2 |
| Season (summer = 1, mid-season = 2, winter=3)^a^ | 6 |
| Sqrt(Traffic in the nearest road) *sqrt(N)* | 1 |
| Industrial land use in 300m buffer *percent area* | 2 |
| Population in 100m buffer *N* | 2 |
| Measurement year *year* | 1 |
| Total length of major roads in 1000m buffer *m* | 4 |
| Temperature *Celsius* | 5 |
| Altitude *m* | 1 |
| Low density residential land use in 200m buffer *percent area* | 3 |
| Boundary layer height *m* | 3 |
| Total length of major roads in 500m buffer *m* | 4 |
| **Urban Model** |  |
| Log (NO_2_ from dispersion model) *log(NO_2_ concentration)* | 3 |
| Log (NO_2_ from AQM Payerne) *log(NO_2_ concentration)* | 7 |
| Sqrt (vehicles in 100m buffer) | 4 |
| Log(1/distance to the nearest major road) *log(1/m)* | 3 |
| Measurement year *year* | 2 |
| Season (summer = 1, mid-season = 2, winter=3)^a^ | 9 |
| Industrial land use in 300m buffer *percent area* | 1 |
| Population in 100m buffer *N* | 2 |
| Total length of major roads in 100m buffer *m* | 4 |
| Temperature *Celsius* | 7 |
| (Boundary layer height)^2 *(m^2)* | 2 |

Note: interaction and quadratic terms are not shown

Table S3. Kappa statistics for External validation – measured vs estimated concentration in quartiles

| **Weighted Kappa: 0.671** | | **Measured quartile** | | | |
| --- | --- | --- | --- | --- | --- |
|  |  | **1** | **2** | **3** | **4** |
| **Estimated quartile** | **1** | 16 | 2 | 0 | 0 |
|  | **2** | 2 | 10 | 2 | 0 |
|  | **3** | 1 | 1 | 7 | 5 |
|  | **4** | 0 | 0 | 6 | 1 |
